# Preparing Distributed Computing Operations for the HL-LHC Era With Operational Intelligence

**DOI:** 10.3389/fdata.2021.753409

**Published:** 2022-01-07

**Authors:** Alessandro Di Girolamo, Federica Legger, Panos Paparrigopoulos, Jaroslava Schovancová, Thomas Beermann, Michael Boehler, Daniele Bonacorsi, Luca Clissa, Leticia Decker de Sousa, Tommaso Diotalevi, Luca Giommi, Maria Grigorieva, Domenico Giordano, David Hohn, Tomáš Javůrek, Stephane Jezequel, Valentin Kuznetsov, Mario Lassnig, Vasilis Mageirakos, Micol Olocco, Siarhei Padolski, Matteo Paltenghi, Lorenzo Rinaldi, Mayank Sharma, Simone Rossi Tisbeni, Nikodemas Tuckus

**Affiliations:** ^1^ CERN, Geneva, Switzerland; ^2^ INFN Turin, Torino, Italy; ^3^ Bergische Universitaet Wuppertal, Wuppertal, Germany; ^4^ Physikalisches Institut, Albert-Ludwigs-Universitaet Freiburg, Freiburg, Germany; ^5^ University of Bologna, Bologna, Italy; ^6^ INFN Bologna, Bologna, Italy; ^7^ Lomonosov Moscow State University, Moscow, Russia; ^8^ LAPP, Université Grenoble Alpes, Univrsité. Savoie Mont Blanc, CNRS/IN2P3, Annecy, France; ^9^ Cornell University, Ithaca, NY, United States; ^10^ Brookhaven National Laboratory, Upton, NY, United States; ^11^ INFN-CNAF Bologna, Bologna, Italy; ^12^ Vilnius University, Vilnius, Lithuania

**Keywords:** distributed computing operations, operational intelligence, HL-LHC, resources optimization, ML, NLP

## Abstract

As a joint effort from various communities involved in the Worldwide LHC Computing Grid, the Operational Intelligence project aims at increasing the level of automation in computing operations and reducing human interventions. The distributed computing systems currently deployed by the LHC experiments have proven to be mature and capable of meeting the experimental goals, by allowing timely delivery of scientific results. However, a substantial number of interventions from software developers, shifters, and operational teams is needed to efficiently manage such heterogenous infrastructures. Under the scope of the Operational Intelligence project, experts from several areas have gathered to propose and work on “smart” solutions. Machine learning, data mining, log analysis, and anomaly detection are only some of the tools we have evaluated for our use cases. In this community study contribution, we report on the development of a suite of operational intelligence services to cover various use cases: workload management, data management, and site operations.

## 1 Introduction

We formed the operational intelligence (OpInt) initiative to increase the level of automation in computing operations and reduce human interventions. In the approaching High-Lumi LHC (HL-LHC) era, we may be granted the computing resources needed to process, analyze, and store an order of magnitude more than the current LHC data. However, the person–power available to operate this infrastructure will not increase at a similar rate, if at all. Therefore, we pursue the outlined automation objectives in two ways: we organize a regular, technical, cross-experiment forum that brings people from various teams together to share ideas, experience, and code, and we develop tools to automate computing operations, exploiting state-of-the-art technology.

The Worldwide LHC Computing Grid (WLCG) (Bird, 2011) project is a global collaboration of around 170 computing centers in more than 40 countries, linking up national and international grid infrastructures. It provides a seamless access to computing resources which include data storage capacity, processing power, sensors, and visualization tools, the resources that are capable to process over two million tasks daily, leveraging over one million computer cores and 1 exabyte of storage. It is a very heterogenous infrastructure. We, as a community, strive to exploit these resources, use the infrastructure efficiently and optimize its usage, and squeeze every available compute cycle out of it. We are passionate about optimizing our software as well as evolving the infrastructure and integrating novel configurations, approaches, and tools, such as compute accelerators, to assure a sustainable future.

Notably, machine learning (ML) models applied to the prediction of intelligent data placements and access patterns can help to increase the efficiency of resource exploitation and the overall throughput of the experiments’ distributed computing infrastructures. Time series analyses allow for the estimation of the time needed to complete certain tasks, such as processing a certain number of events or transferring a certain amount of data. Anomaly detection techniques assist with prediction of system failures. Natural language processing (NLP) techniques can be used to analyze logs of services and extract information to automatically assist the operators; each year, thousands of tickets are submitted to ATLAS (ATLAS Collaboration, 2008) and CMS (ATLAS Collaboration, 2008) issue-tracking systems and further analyzed by the experiment operators. Analysis of the operators’ actions is used to automate tasks such as creating support-requesting tickets to support centers or to suggest possible solutions to recurring issues. Some of those efforts that were born out of the discussions are already being used to reduce operational costs.

In this article, we cover operational intelligence areas that focus on optimization of computing infrastructure, site operations, and workflow management and data management operations. We summarize activities that have been delivered to production and are already assisting with the automation objectives as well as those that are still in the prototyping phase, the fruits of which will only be harvested in the future.

The structure of the article is as follows: in Section 2, we provide an overview of areas of interest of the operational intelligence activities, detailing various activities in different stages of completeness, from conceptual design ideas to those that have already been brought to production. In Section 3, we summarize directions for the future evolution. Section 4 provides conclusions of the OpInt activities and plans.

## 2 OpInt Areas of Interest

One of the most important aspects of the OpInt project is enabling the collaboration between the different experiments and teams that take part in it. High-energy physics computing is a complex and diverse world, but many components used in software development can be shared across projects. As we are a diverse community, some of the activities aim toward modest evolution and harmonization, rather than revolution, with a highly visible impact on users. Nevertheless, one of the challenges we are facing as we approach the HL-LHC era is the nurturing of cross-team, cross-experiment, and cross-domain collaborations, to share ideas and tools, working toward a common goal, together, making our community stronger and sustainable on the long term. In the following paragraphs, we summarize the areas of the challenges that we are addressing.

### 2.1 Monitoring the Computing Infrastructures—Tools and Their Unification

The OpInt activity heavily relies on the monitoring infrastructure that consists of experiment-agnostic and specific components described later in this section. One of the challenges we address is finding the correct balance of generalization versus customization in the infrastructure configuration, in the tools, the data formats, interfaces, access protocols, and visualization. We believe it is important to offer a shared development platform for all the OpInt-related projects in order to foster code re-usability and enable a quick prototyping of new ideas by using pre-created components. Further description of the monitoring infrastructure is given in Section 2.1.

### 2.2 Predictive and Reactive Site Operations

Predictive maintenance is a sought-after topic in a variety of applications to complex systems, aiming at identifying maintenance tasks, cutting operational costs, and boosting overall productivity. It is based on the identification of the system state, from diagnosis to prediction, involving decision-making activities to autonomously control the system itself. As such, it also works for computing centers and data centers as well as data infrastructures. The health maintenance of the computing systems can be divided in at least three big activity areas: 1) diagnosis, 2) prevention, and 3) prediction (Decker de Sousa et al., 2019). This activity concerns the first step of the maintenance, performed using anomaly detection solutions. Anomaly detection is a chosen strategy to feed a diagnostic system. In this context, it refers to preliminary data treatment to evaluate the current system status using scores, variables, or metrics extracted from log data. We describe the anomaly detection approach in Section 2.2, the predictive site maintenance in Section 2.3, and dynamic, reactive job shaping in HammerCloud in Section 2.4.

### 2.3 CMS Intelligent Alert System

The intelligent alert system for the CMS experiment, further described in Section 2.5, aggregates alerts from different experiment-specific monitoring systems and augments them with additional information from the multi-experiment monitoring tool Site Status Board (SSB) (Andreeva et al., 2012a) and Global Grid User Support (GGUS) (Antoni et al., 2008). The GGUS is a ticketing system used in the HEP-distributed computing domain (and others too) that facilitates a standardized issue-reporting and communication interface among the experiment teams (e.g., operators, experts, developers, and users) and the sites. In the CMS intelligent alert system, focusing mainly on network and database interventions and outages, annotations are created to assist the experiment’s operators.

### 2.4 Workflow Management—Jobs Buster

The Jobs Buster is an application that assists with spotting job failures and chasing their root causes in a timely manner. It is built with the OpInt development platform, comparing features of successful and failed jobs taken within the same time frame. The statistics of failed and successful jobs are used to train a failure model. The Jobs Buster is described in Section 2.6.

### 2.5 Error Messages Clustering

Clustering of error messages is a possible way to simplify the analysis of error messages encountered in large-scale distributed systems. With the considerable quantity of failures, the variety of sources and types of errors, and considering the unpredictable textual patterns of error messages, the traditional method of manually reviewing errors is impractical.

The error message clustering employs the following categorization: messages encountered only once or several times are considered as anomalies, whereas messages with the same textual pattern and error conditions are grouped together. Groups of similar messages are then described by the common textual patterns and keywords. All messages are linked to the sources, and messages may be of various types and from different sources. The error message clustering is described in Section 2.7.

### 2.6 FTS Logs, Errors, and Failures Analysis

The distribution of the large volumes of data collected by the LHC is mainly managed by the file transfer service (FTS) (Karavakis et al., 2020). Each data transfer operation among grid sites is tracked by FTS, and the related log files contain large volumes of information related to performance and errors. Analysis of the FTS logs represents a typical OpInt workflow, processing logs of the FTS service with a ML model and visualizing the clusters and their updates in a monitoring dashboard. It is written in an experiment-agnostic manner (developed in the CMS experiment), and one of the future challenges will be to integrate it with the infrastructure of another experiment (notably the ATLAS experiment).

Analysis of FTS errors and failures represents a challenge to distributed data management (DDM) infrastructure operations. It is performed by teams of trained shifters and thus represents one of the challenges for OpInt: how can we improve the operations automation, in order to benefit from the expertise of the shifters elsewhere in the community? We have adopted an unsupervised learning approach to minimize the shifters’ effort and to enable the discovery of new failure patterns related to FTS transfers.

A further description of the analysis of FTS errors and failures is presented in Section 2.8.

### 2.7 NLP Applications in Rucio

Rucio (Barisits et al., 2019) is an open-source project for managing large amounts of data, and it is now adopted by many communities. Rucio exploits NLP techniques to automate the processing of user support requests coming from a variety of support channels, up to a certain complexity of the request, and provides a collection of information to the user-requesting support. A further description of NLP applications in Rucio is provided in Section 2.9.

### 2.8 Monitoring the Computing Infrastructures

In this section, we describe the infrastructure that the OpInt activity heavily relies on, with the experiment-agnostic and specific components.

### 2.9 CERN MONIT Infrastructure

The CERN IT department offers a stack of monitoring services, referred to as MONIT (Aimar et al., 2017), based on different open-source technologies, supporting a variety of data sources. Clients can either actively push data to MONIT or pull it. All the collected data are processed by Apache Kafka at the MONIT core, with Apache Spark used to enrich and aggregate the data (Apache Software Foundation). Data are written into three different data stores: InfluxDB^1^ for time-series data, ElasticSearch^2^ for more detailed data with a limited retention, and Apache Hadoop (Apache Software Foundation) for long-term archival. Data visualization is offered via Grafana^3^ dashboards.

### 2.10 ATLAS Monitoring Infrastructure

The ATLAS monitoring infrastructure (Beermann et al., 2020) is based on MONIT. The main components are the DDM transfer monitoring and accounting, the jobs monitoring and accounting, and the SSB. The DDM dashboard is based on the Rucio events and traces, which are collected from Apache ActiveMQ (Apache Software Foundation). They are enriched with topology information from the Computing Resource Information Catalogue (CRIC) (Anisenkov et al., 2020), aggregated and written both to InfluxDB and ElasticSearch. The DDM-accounting dashboards are based on preprocessed accounting reports from Rucio, enriched with topology information and written to ElasticSearch. The jobs monitoring and accounting dashboards data are collected directly from the PanDA (Maeno et al., 2014) database, processed in Apache Kafka and written into ElasticSearch. Finally, the SSB is a meta-dashboard that collects data from a variety of different sources, such as DDM and jobs monitoring, SAM tests (Andreeva et al., 2012b), and GGUS tickets, and presents the aggregated data to easily spot a site issue.

### 2.11 CMS Monitoring Infrastructure

CMS benefits from MONIT, and its own monitoring infrastructure (Ariza-Porras et al., 2021), which is hosted on a set of Kubernetes (k8s) clusters (Kubernetes, 2021). The CMS monitoring cluster provides access to common infrastructure monitoring services, interfacing their output with smart-alerting and notification applications, for example, the CMS intelligent alert system, providing a uniform real-time messaging and alerting infrastructure that assists the operators in their decision-making and escalation processes. Currently, the CMS monitoring infrastructure scrapes metrics from hundreds of computing nodes, runs 125 exporters, and provides more than 3,000 measurements as well as hundreds of different alert records and alert rules. It stores around 500 billion data points with a data retention policy corresponding to the last 30 days.

### 2.12 OpInt Framework

In order to share expertise and re-use code and approaches across the board, we started developing the OpInt framework^4^ and have already created the following components:

• Abstract data fetchers to enable getting data from various sources, and a demo implementation of a fetcher that gets data from an Apache Hadoop endpoint.
• A module to schedule and execute data-fetching tasks either periodically or on demand.
• A module to construct REST APIs to share processed data with other services.
• database integration to store fetched and aggregated data.
• An abstract template for NLP-processing pipelines. Two of the pipelines that are used in our projects have already been adapted to follow the abstract implementation.
• A sample application that can be used to kick-start new projects.

The OpInt framework has already been utilized to run the Jobs Buster application described in Section 2.6. Migration of other existing projects is ongoing. Some next steps have been identified, including the refinement of the framework and consolidation of the deployment method, for example, in a Kubernetes cluster. Future developments will focus on a feedback mechanism implementation, for our users to provide feedback on our results, to improve the quality of the information exposed to the operator/user. The feedback mechanism will be flexible to meet the needs of each individual application. A generic authentication and authorization component to enable login through CERN Single Sign-On and other sources will be implemented in the near future.

### 2.13 Anomaly Detection

The detection of anomalies in data center metrics using machine learning algorithms is an active field of development, with promising advantages in terms of increased quality of service and cost efficiency. The most important players in the field, namely large commercial data centers, often prefer to keep their research findings undisclosed to increase their competitive advantage. The WLCG community has started several initiatives to tackle this subject and fill this gap. One such example is a modular anomaly detection system, developed with open-source technologies, which can be easily adopted and/or adapted by other data centers and service managers (Giordano et al., 2021). Besides the data engineering task, the work also involves data science tasks, including mathematical formulation of the anomaly detection problem for time series and the implementation of multiple algorithms with both traditional and deep-learning methods. In addition, the need of field-specific annotated datasets is highlighted, and a straightforward solution based on Grafana annotations is proposed in order to collect them. The solution extends the Grafana annotations tool to add two buttons (“Normal” and “Anomaly”) and to associate tags with events.

### 2.14 Predictive Site Maintenance

Log data are often unstructured, in which the format and semantics may vary significantly from system to system, making an approach toward a general-purpose log-based anomaly detection system extremely challenging. Supervised approaches provide more accurate results at the cost of requiring a large dataset of labeled entries, which is a hard restrictive requirement in the majority of real-world scenarios. Generally, labeled anomalous logs are not available and because of this supervised approaches are seldom used without expensive data pretreatment. In addition, to generate *ad hoc* solutions for each existing system is counterproductive since a single data center can incorporate hundreds of computing sub-systems. For this reason, general-purpose solutions are preferable even if, initially, they are expected to perform worse than dedicated solutions.

We are exploring feasibility of isolation forest (IF) techniques in the area of predictive site maintenance. The IF technique is a machine learning algorithm commonly applied to anomaly detection problems, for example, to identify anomalous samples in unlabeled data (Liu et al., 2008). A novel IF log–based anomaly detection approach has been proposed (Farzad and Gulliver, 2020), focusing on identifying normal log samples instead of anomalies to extract features through an autoencoder network. A One-class Support Vector Machine classifier is used to identify anomalous time-windows, in which the classifier is based on the logging activity and volatility extraction from the unstructured log data (Minarini and Decker, 2020).

NLP techniques stand out as one of the most popular classes of strategies. Automated log parsing offers promising results through standard off-the-shelf NLP algorithms (Bertero et al., 2017). Further improvement is possible (Wang et al., 2020) by applying word2vec (Mikolov et al., 2013) algorithms to log events. Whilst log parsing is often used to improve the effectiveness of anomaly detection tools, NLP is a very expensive technique since it was originally envisioned to approach a much more complex class of problems, such as handling natural language. The OpInt effort is focused on NLP solutions.

### 2.15 HammerCloud Job Shaping

The functionality of the compute sites of the WLCG for the ATLAS and CMS experiments is verified by a large number of experiment-specific test jobs. These jobs are steered, controlled, and monitored by the HammerCloud (Schovancová et al., 2019) testing infrastructure. ATLAS HammerCloud runs different functional tests, continuously checking the site status by representative MC simulations (PFT) and analysis jobs (AFT). If these test jobs fail, the site is automatically excluded from the central ATLAS workflow management system (WFMS): only test jobs will be sent to the site until the test jobs succeed again. Thanks to this auto-exclusion mechanism, the success rate of the user jobs is improved, since jobs are then only sent to properly working sites.

**FIGURE 1:**
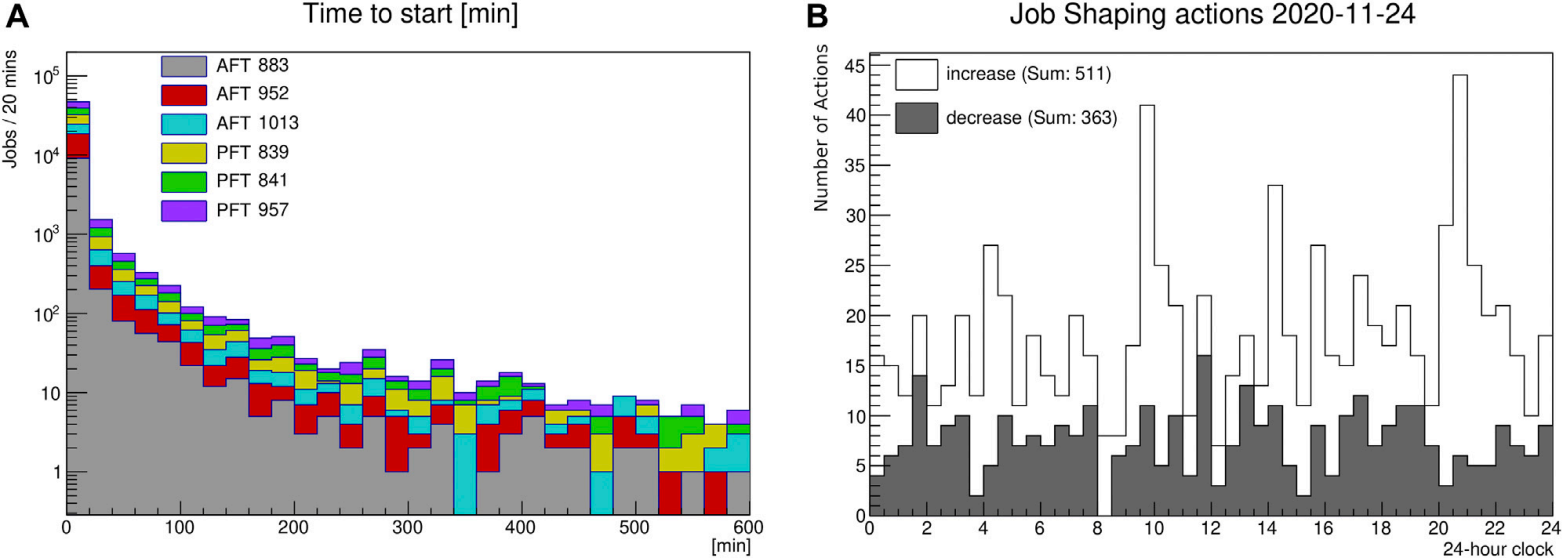
**(A)** Time-to-start in minutes of HammerCloud functional test jobs used for auto-exclusion and re-inclusion into the ATLAS Grid WFMS. **(B)** Example histogram from 2020-11-24. Number of job shaping actions every 30 min (empty: increase; filled: decrease) of the parallel running test jobs.

With job shaping, we aim to speed up auto-exclude and recovery decisions made by HammerCloud. This will be achieved by dynamically adjusting the frequency of test jobs based on the latest test job results. Since the auto-exclude decision is based on several functional test jobs processed on a given site, a delay of any individual job can delay the decision as a whole for the site. Therefore, we have analyzed the average run time and the time to start for all test jobs used for auto-exclusion over a week on all sites. The average run time is well below 30 min, which is following the requirements of efficient functional tests: they should be fast and lightweight in terms of resource utilization. The time-to-start peaks below 20 min, but it also has a long tail up to more than 10 h, as shown in (Figure 1A). Based on these numbers, a 2 -hour run time threshold has been defined.

In order to speed up the decision time in case of jobs remaining in the state “submitted - waiting to start”, for example, if the current job is just stuck, increasing the number of parallel running test jobs would improve the situation. This “submitted - waiting to start” clogging may be a result of a queue mechanism mis-configuration or high demand for capacity at the site serving demands of multiple experiment in a shared environment, the central job scheduler of the experiment may have only a little wiggling space to attempt to unclog the site; one of the approaches is to avalanche more probing jobs, as the job shaping mechanism does. At the same time, in order to avoid wasting compute resources for unnecessary test jobs, the job shaping mechanism adjusts the number of parallel running jobs dynamically: in case of missing job results, five additional jobs per test type are submitted, and as soon as the tool recognizes jobs which have been finished within the last 2 hours, the number of parallel running jobs is decreased to 1 again.

As shown in Figure 1B, the job shaping tool triggers an order of 1,000 increase and decrease actions per day. Considering the different AFT test jobs running on 150 sites and PFT jobs on 200 sites, this translates into roughly one job shaping action per site and one test type per day. The increase and decrease decisions follow a mainly uniform distribution. The isolated spikes which can be seen at 10 a.m., 2 p.m., and 8:30 p.m. are caused by the same set of faulty sites. The increase actions are triggered in three different periods, since the underlying tests have been started at 8 a.m., 12 noon, and 6:30 p.m., respectively. In order to quantify the improvement of the job shaping, we have analyzed the number of sites missing test jobs for the auto-exclusion and re-inclusion decision in the month before enabling job shaping, compared to 10–11 sites afterward. On average, there were 12–14 sites with missing test jobs before enabling job shaping, compared to 10–11 sites afterward.

### 2.16 CMS Intelligent Alert System

Within the CMS experiment, we developed an intelligent alert system to automate our dashboard with SSB and GGUS alerts. The system is based on the Prometheus and AlertManager RESTful APIs to fetch, create, and update new or existing alerts and supplement them with additional information obtained from SSB and GGUS services.

First, we developed a general purpose scraper for the GGUS system by fetching its tickets via HTTP end-point in the CSV format. Then, we used CERN MONIT ElasticSearch instance to obtain SSB alerts.

Finally, our system processes this information and constructs necessary annotations on a set of dashboards. All parameters are configurable: from various HTTP end-points to regular expressions used in alert matching as well as dashboard tags.

We deployed this service in the CMS monitoring cluster, and it is integrated with our Prometheus and AlertManager services. So far, we annotate dashboards related to cmsweb services, which provide service overview for the experiment’s compute services. The current matching expression watches for network and database interventions and outages as well as issues related to the CERN MONIT infrastructure. The annotations are applied on a tag-matching basis, i.e., we put specific tags on our dashboard and use them for HTTP request annotations.

### 2.17 Jobs Buster

Jobs Buster is an application built within the OpInt framework to detect large groups of job failures and identify the most probable cause. We used a CatBoost library (Prokhorenkova et al., 2019) and the CatBoostClassifier to train a gradient-boosted tree, which were able to predict the outcome status of a job by its features. The CatBoost library provides embedded support of categorical variables and efficient learning. Using a model previously trained on a particular data slice, Jobs Buster extracts the list of features with importance above a threshold of 0.5 and performs a ranking of its values. Groups of feature-value pair sets for failing jobs define clusters that might be identified with a specific source of failure (a particular task fails on a selected grid site due to misconfiguration or some rack in a data center is out of order).

Jobs Buster uses eight features that describe a job, such as the site, the user who submitted a task, and the number of required CPU cores. It also preprocesses the text into diagnostic messages, constructing a corpus of words from all words in all messages for a particular time window. When performing the frequency analysis, a custom list of stop words is excluded. All diagnostic messages of a particular job are converted into a hash and then merged into one string. Once each job descriptor has this concatenated diagnosis string, all failed jobs are split into groups by the diagnosis value, and we train models within each group separately as described previously. This tool is continuously assessing jobs from the ATLAS experiment.

### 2.18 Error Messages Clustering

Analysis of error messages encountered in large-scale distributed systems has become one of the crucial tasks for the monitoring of computing resources. The monitoring systems utilize error messages to *detect anomalies* (failures that happened only a few times in some period), to *identify duplicated issues* (in practice, issues tend to recur many times), to *diagnose failures* (to discover the resource, user, or other entity related to messages indicating some issue), and to *analyze failures retrospectively*.

There is already a variety of tools for log and error message parsing that perform clustering using methods such as frequent pattern mining, machine learning clustering, grouping by longest common subsequence, heuristics, parsing trees, and evolutionary algorithms (Zhu et al., 2019). But, the existing tools have some limitations: most of them require preprocessing, most are customized for specific data, and they do not allow error messages to be linked with other entities, meaning messages cannot be clustered together with auxiliary data.

We are developing an error messages clustering framework that consists of several stages: data trimming, vectorization, clustering, and clusters description. Data trimming—cleaning, tokenization, and regrouping of data—allows reduction of the initial number of messages by about 90–95%. Error messages are split into tokens, cleaned from insignificant substrings (for the clusterization), and regrouped by the equal cleaned patterns. The vectorization stage is based on the word embeddings technique: at the beginning, each token (or word) is converted to a numerical vector using the word2vec method, and then the average vector for each error message is calculated. At the clustering stage, we intend to explore various machine learning or statistical algorithms to cluster the numerical vectors, such as DBSCAN, HDBSCAN, Optics, Hierarchical, and k-means. Some of these algorithms are density-based as they do not require the initial knowledge of the number of clusters and allow the detection of anomalies. For the DBSCAN and Hierarchical algorithms, the epsilon parameter (intra-cluster distance) is selected automatically based on the data. k-means can be used additionally if we need the deterministic output. The accuracy of the clustering highly depends on the quality of the word2vec model. After training on a large volume of error messages, it becomes the basis for the mapping of error messages to numerical vectors. Larger models can achieve better accuracy in clustering tasks. The clusters description stage searches for common textual patterns and common key phrases for all messages in a cluster. For this purpose, we evaluate the performance of the Levenshtein similarity matching and various keyword extraction algorithms, such as RAKE, TopicRank, and YAKE.

### 2.19 Analysis of FTS Errors and Failures

One of the main concerns in data transfer operations is to promptly detect and solve issues that affect the functioning of the infrastructure. On our way toward improving automation of DDM operations, we adopted an unsupervised learning approach to minimize experts’ effort and enable discovering new failure patterns related to FTS transfers. This tool targets the operators and aims to assist with understanding the infrastructure issues in the data transfer area.

The pipeline consists of two main steps: *1*) vectorization and *2*) clustering. In the *vectorization step*, we concatenate the raw error string with source and destination hostnames, and we use a word2vec model that learns how to map all that information to a vectorial space of a given size, where similar errors are expected to be close together. This is to transform the textual information into a convenient numeric representation and serves as preprocessing for the next steps. A *clustering algorithm* is the applied to get groups of related errors: we pre-train a word2vec model on a big dataset and run a k-means++ algorithm (Arthur and Vassilvitskii, 2007) online during the monitoring shifts.

**FIGURE 2:**
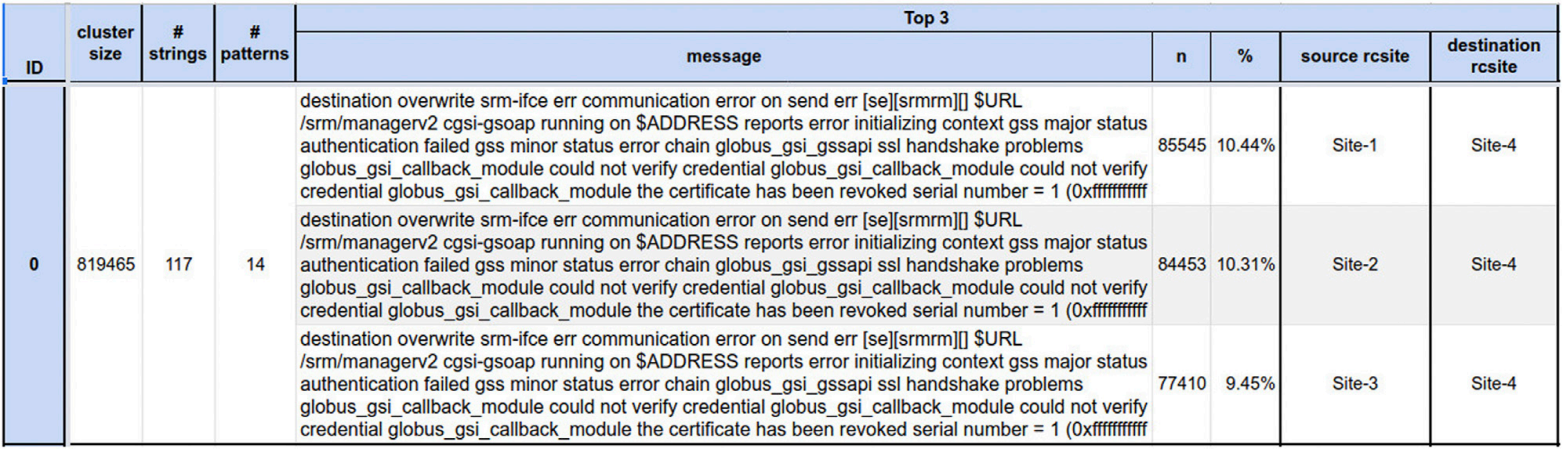
Example of an error message cluster summary.

In order to demonstrate the approach, we report an analysis of FTS data from one full day of operation. Figure 2 shows an example of a summary table for the biggest cluster found by the model.

**FIGURE 3:**
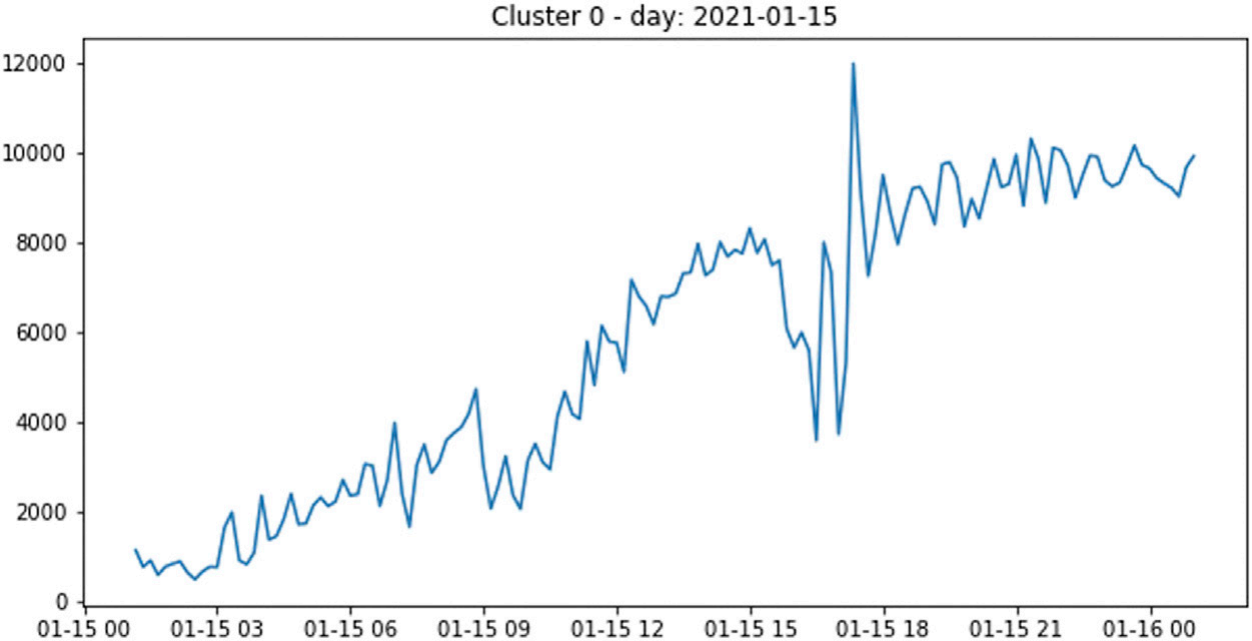
Time evolution of cluster 0: the plot shows the count of errors in bins of 10 min.

The first three columns provide numeric summaries: 1) the cluster size, 2) the number of unique strings within the cluster, and 3) the number of unique patterns: unique strings after the removal of parametric parts such as paths, IP addresses, and URLs. The model learns to *abstract* message parameters and to group strings that are similar except for the parametric parts. As a result, the initial amount of errors is reduced to a number of patterns which is lowered by several orders of magnitude. The core part of this visualization is then represented by the top 3 sections, where the most frequent triplets of pattern, source, and destination sites are reported in a descending order, together with their multiplicity and the percentage over the cluster size. There, we extract several insights, for example, whether a pattern is responsible for a large number of failures or if it accounts for a conspicuous fraction of the cluster. In addition, one can investigate the contribution of source/destination site pairs, as in Figure 2, where Site-4 clearly seems to have a problem as destination. Another useful piece of information given by the cluster’s time evolution plot (shown in Figure 3) is that it can give an immediate indication of whether the problem is transient or not.

Overall, the idea is for the shifters to look at summary tables and time plots for each of the clusters detected by the algorithm, which act as suggestions of possible issues to investigate further, or later, would create automatic alerts, notifications, or even issue reports. The process described previously can be fully automated after tuning model hyper parameters based on metrics, such as average silhouette width score and within-cluster sum of squared (Euclidean) distances, that measure how compact and separated the clusters are. We have conducted an extensive testing as pre-validation comparing the clusters obtained with this approach against GGUS tickets, showing a reasonable overlap between suggested and reported issues. Although it makes sense to cross-check clustering results with tickets, this comparison has some drawbacks. In particular, the procedure is very sensitive to the choice of the time window. It requires a manual check of the ticket information and the cluster content, which makes the comparison lengthy and not scalable.

### 2.20 NLP Applications in Rucio

Data in Rucio is organized using data identifiers (DIDs), which have three levels of granularity: files, datasets, and containers. Datasets and containers are used to organize sets of files in various potentially overlapping groups and to facilitate bulk operations such as transfers or deletions. Users are permitted to perform certain actions on the DIDs: downloads, uploads, or transfers. Due to the large sizes of the collaborations, the experts support a noteworthy amount of requests daily. To reduce the required efforts, we are looking into methods to assist the support team in answering these requests. Ideally, the support would be provided by an intelligent bot able to process and understand the user’s requests and, if possible, trigger appropriate automated action.

The NLP assists in processing questions from users, where the objective is to provide satisfactory answers. A prototype bot capable of handling user’s requests up to a certain level of complexity has been developed, acting with emails as inputs. When a new email is received, it goes through the QuestionDetector, and a question object is created. Originally, the support bot was meant to answer incoming support emails. After an initial data analysis, we expanded that idea and introduced new data sources and the option to query the bot directly. A question from a user is treated as a query by the search engine in the next step. Once a question has been detected from a new email or given by a user query, the search engine looks into the support bot data storage and retrieves the most similar documents. Documents are archived questions from emails or GitHub issues and Rucio documentation pages from GitHub as well as FAQ question–answer pairs created by Rucio experts. The search engine subclasses exist for each data source. Once the most relevant documents have been retrieved, we use them as context for the new question in a SQuAD-like (Rajpurkar, 2021) manner to generate answers with a given confidence. The current implementation caches and uses DistilBERT (Sanh et al., 2020) and BERT large-cased/uncased models, fine-tuned on the SQuAD dataset.

The NLP applications described in this section are in different maturity states, from prototypes to production deployment. They aim to reduce human person–power cost in DDM operations, targeting partly DDM operation experts, partly suggestion, and chat systems for the user community.

## 3 Future Developments

We foresee activities developing in a variety of areas:

• Predictive Site Maintenance: We foresee tuning of anomaly detection approaches applied to data centers’ metrics as well as further exploration and exploitation of granular computing classifiers in predictive site maintenance. We intend to introduce specialized debugging tests to probe further the root cause of the failure with HammerCloud dynamic job shaping, and provide suggestions to the operators, or even assist in the automation of the issue reporting and mitigation.
• Jobs Buster: We plan to add long-term models into Jobs Buster, enriching jobs features with information extracted from failures that occurred in the past.
• Error Messages Clustering: Further framework developments involve code parallelization of the time-expensive stages.
• FTS Logs, Errors and Failures Analysis: We wish to build a reference dataset to store labels for error categories, root causes, priority, and solving actions, assisting in performance optimization. Additionally, further NLP tools such as Question Answering or Named Entity Recognition (NER) look promising to support our target of understanding the root causes and suggesting solving actions for the issues.
• NLP Applications in Rucio: Creating a user interface will allow us to deploy the support bot on a test server to a wider set of users, expanding the reach from the beta-testers and from the developers and experts community to the user communities. Their questions and the answers given can then be supervised and examined to create a dataset for the fine-tuning of the models. Additionally, we wish to create a NER tagger to detect Rucio-specific language entities, exploring potential to boost the tool performance significantly, and provide a way to create dynamic answers.

## 4 Conclusion

The operational intelligence initiative brings together experts from various WLCG communities and is aiming to reduce the person–power cost of operating distributed computing systems. We presented an overview of activities in varying stages of completeness in the areas of computing centers operation and the workflow and data management, which represent only three of the areas, where innovative approaches can bring substantial improvement while we benefit from the state-of-the-art technologies.

The projects described in Section 2 and Section 3 are summarized in Table 1. In the collaborative spirit, in compliance with a variety of computer security and data privacy guidelines and policies applicable to our environment, we have been sharing the code developed in the scope of the various operational intelligence initiative projects in a GitHub repository^5^.

**TABLE 1:** List of OpInt projects deployed in production and under development, with their current status.

Project	Status	Detail
Intelligent alert system	In production in one experiment; concept in development in another one	Section 2
Jobs Buster	In production in one experiment	Section 2.6
FTS log clustering	In production in one experiment; concept in development in another one	Section 2.7
HammerCloud job shaping	In production in one experiment	Section 2.4
Shared k8s cluster	Infrastructure deployed, wider adoption by two experiments in progress	Section 2.1
Cloud anomaly detection	Infrastructure and algorithms prototyped, commissioning in progress	Section 2.2; (Giordano et al., 2021)
FTS anomaly detection	Prototype developed in one experiment; generalization and adoption by another experiment in progress	Section 2.8
Predictive site maintenance	Code in development	Section 2.3

Although some of the projects have recently entered only the prototyping phase, some are in the development and commissioning phase and some of the activities are already in production and bringing fruits in terms of saving person–power, already now: the intelligent alert system, combined with the monitoring cluster in CMS, and the Rucio NLP applications as well as Jobs Buster and HammerCloud job shaping in ATLAS.

The combined monitoring and alerting infrastructure is a fully automated system. It consists of five Kubernetes clusters managed by less than two FTEs (Full-time equivalent). It provides an enormous amount of information to the collaboration. The technology allows to automate many operations, from service deployments (via continuous integration/continuous deployment processes) to dashboard annotations (via OptInt modules). The use of common monitoring tools and technologies allowed the experiment to reduce the number of custom, in-house developed applications that would require the allocation of additional FTEs for development and maintenance. The automated service maintenance via a common set of alert notifications (i.e., importance of the notification is assessed by the system, prior to escalating to a human operators) provides much better insight into operations, allowing operators to timely address the most important and urgent issues.

Rucio NLP applications assist with repetitive tasks in the area of DDM operations, particularly targeting the support of the user communities. The Jobs Buster provides insights into jobs failure causes in ATLAS, as it extracts the essential information from the jobs’ logs, and serves it in a comprehensive manner to the operators. The HammerCloud job shaping tool assesses the job failures of test jobs and avalanches a set of probing jobs to collect and abstract further information about the underlying cause of the failure.

Since the beginning of LHC data taking (and even before that), digging into plethora of logs spread across different tools and servers, with different format to present the log information, and abstracting the essential information about the system failure, whichever system or systems they were, has been the core activity of the human operators, including the experts. It would account for at least 80% of the operators’ work time. Having automation tools such as Jobs Buster and HammerCloud performing the laborious steps of the failure chasing saga instead of the human operators is a great achievement and still have potential for automation that we are exploring as a next step, with the planned automated reports (e.g., GGUS tickets to the sites), describing the underlying cause and suggesting which corrective actions should have been taken. These tools not only reduce the time operators need to spend to identify potential issues but also help to increase the utilization of computing resources.

The established procedures of large experiments are well-understood and trusted, such that integrating new ideas and approaches, even general ones, requires significant time and careful integration and validation. The complex heterogenous WLCG infrastructure represents a stimulating environment to apply data science techniques to assist in pushing forward the frontiers of fundamental science, and the initial objectives achieved supported by the positive feedback from the community confirm that integrating further projects into OpInt pipelines is a step in the right direction, demonstrating that collaboration across the table is not only possible but also fruitful, and it is one of the ways to make our community sustainable and capable of addressing the future challenges.

## Data Availability

The data analyzed in this study is subjected to the following licenses/restrictions: log files of distributed computing services to which CERN Operational Circulars apply, in particular CERN OC11. Request to access these datasets should be directed to AG, Alessandro.Di.Girolamo@cern.ch.

## Footnotes

1
https://www.influxdata.com/.

2
https://www.elastic.co/.

3
https://grafana.com/.

4
https://github.com/operationalintelligence/opint-framework.

5
https://github.com/operationalintelligence/.
